# Functional characterisation of human cells harbouring a novel t(2p;7p) translocation involving *TNS3* and *EXOC6B* genes

**DOI:** 10.1186/1471-2350-14-65

**Published:** 2013-06-28

**Authors:** Desiree Ludwig, Jessica Carter, James R Smith, Giuseppe Borsani, Sergio Barlati, Sassan Hafizi

**Affiliations:** 1 Institute of Biomedical and Biomolecular Science (IBBS), University of Portsmouth, School of Pharmacy & Biomedical Sciences, St. Michael’s Building, White Swan Road, Portsmouth PO1 2DT, UK; 2 Dipartimento di Scienze Biomediche e Biotecnologie, Sezione di Biologia e Genetica, Universita' di Brescia, Brescia, Italy

**Keywords:** Tensin, Exocyst, Translocation, Chimera, Haploinsufficiency, Cell migration

## Abstract

**Background:**

Tensin3 is an intracellular cytoskeleton-regulating protein, the loss of which is associated with increased cell motility, as has been observed in some human cancers. A novel chromosomal translocation, t(2;7)(p13;p12), present in a patient with a complex syndromic phenotype, directly involves Tensin3 (*TNS3*) and *EXOC6B* genes. This translocation could impair the expression of Tensin3 and ExoC6B proteins, and potentially produce two novel fusion transcripts. In the present study, we have investigated the expression and phenotypic features of these potential products in cultured cells from the proband.

**Methods:**

Skin fibroblasts isolated from the proband as well as an age-matched control were grown in cell culture. Cells were used for quantitative RT-PCR, western blot and immunofluorescent confocal microscopy, which determined Tensin3 gene and protein expression. Phase-contrast and confocal microscopy additionally revealed cellular phenotype differences. A scratch wound assay monitored by live cell imaging measured cellular migration rates.

**Results:**

The levels of Tensin3 at both mRNA and protein levels were lower in proband cells versus control fibroblasts. Proband cells displayed broader and shorter morphologies versus control fibroblasts, and immunofluorescent staining revealed additional Tensin3 expression along cytoskeletal filaments and the cell periphery only in control fibroblasts. In addition, proband fibroblasts showed a significantly higher migration rate than control cells over 24 h.

**Conclusions:**

The phenotypic changes observed in proband cells may arise from *TNS3* haploinsufficiency, causing partial loss of full-length Tensin3 protein. These results further expose a role for Tensin3 in cytoskeletal organisation and cell motility and may also help to explain the syndromic features observed in the patient.

## Background

The Tensins are a family of intracellular proteins, including Tensin1, -2, -3 and −4 (cten), that appear capable of linking the cytoskeleton to the cytoplasmic tails of some transmembrane receptors, including β integrins [1] and receptor tyrosine kinases [2,3]. Tensins are composed of a characteristic set of domains that are involved in discrete protein-protein interactions. The N terminal is believed to bind F-actin whilst the C-terminal houses a tandem pairing of SH2 and PTB domains, which are binding interfaces for several interaction partners including FAK [4], p130Cas [3] and the tumour suppressor DLC-1 [5]. Through these domain-mediated interactions, the Tensins are believed to regulate cytoskeletal dynamics and thereby related processes such as cell shape, process formation, motility and apoptosis [6].

Therefore, any possible aberration that results in an altered functionality or a complete or partial loss of the Tensin protein, may feature in certain pathologies. This has been shown to be the case in several cancers, where Tensin genes show aberrant expression, with Tensin2 and Tensin3 [7,8] being down-regulated whilst Tensin4 (cten) is conversely upregulated [9,10]. A mechanistic explanation for these conflicting observations is that Tensin4, which is a shorter Tensin variant and lacks the N-terminal actin-binding domains, displaces the longer Tensins [11]. This impairs the tethering of the actin filaments to the plasma membrane and consequently alters the cytoskeletal makeup of the cell, resulting in loss of cell shape and increased cell motility. Such a phenomenon may therefore underlie the metastatic potential of malignant cancer cells.

We recently identified a unique genetic abnormality involving Tensin3 in a patient who possessed a novel karyotype, resulting from a reciprocal balanced chromosomal translocation, t(2;7)(p13;p12) [12]. The proband as an infant presented with an underdeveloped left kidney and right kidney dysfunction, as well as neutropenia and developmental problems. The proband’s karyotype featured a fusion of chromosomes 2 (contains the *EXOC6B* gene) and 7 (contains the *TNS3* gene), yielding two possible fusion sequences. The *EXOC6B-TNS3* sequence contains exon 1 of the *EXOC6B* gene and the last 15 exons of the *TNS3* gene, whilst the *TNS3-EXOC6B* sequence contains exons 1–15 of the *TNS3* gene and the last 20 exons of the *EXOC6B* gene. ExoC6B (also known as Sec15B, Sec15L2) is an 80–95 kDa protein that is part of the exocyst multi-protein complex, which is involved in the polarised exocytosis process, and also important for other processes such as actin-based membrane protrusion, cell migration, cell communication, cell growth and cell polarity [13]. Therefore, given the normal functions of Tensin3 and ExoC6B, the clinical features observed in the proband in our initial study could have arisen as a result of either haploinsufficiency of the *TNS3* and *EXOC6B* genes and/or expression of dysfunctional chimeric proteins. However, at the time we did not investigate whether both fusion transcripts could potentially give rise to translated protein products due to lack of specific tools for studying either protein.

We gained an initial insight into the molecular features of this homozygous aberration by detecting a marked reduction in expression of Tensin1 in cultured fibroblasts from the proband compared to control ones [12]. Nevertheless, the actual fate of the direct molecular targets of such a cytogenetic perturbation, i.e. Tensin3 and ExoC6B, has not been investigated. Therefore, in the present study, we have continued our investigation on the proband’s cells, and here report our findings on: (i) the state of *TNS3* gene and protein expression, (ii) the potential presence of Tensin3-ExoC6B fusion proteins and (iii) the effect of the altered cytogenetic profile on cell migration of such cells. Our results indicate that partial loss of full-length Tensin3 may be responsible for the phenotypic alteration of the proband’s cells, as observed through enhanced cell migration.

## Methods

### Cell culture

Skin fibroblasts were obtained from the original proband and an age-matched healthy counterpart, as previously described [12]. Written informed consent for participation in the study was obtained from the parents of the proband. Furthermore, the use of the cells for this study was in accordance with the regulations of the ethics committee of the University of Portsmouth. Cells were cultured in Dulbecco’s Modified Eagle Medium (DMEM) supplemented with 20% foetal calf serum, 100 U/ml penicillin, 100 μg/ml streptomycin and 2 mM L-glutamine (Lonza, Slough, UK). Cells were maintained by incubation at 37°C in a humidified atmosphere of 5% CO2 in air and passaged sparingly once a week.

### Real-time quantitative reverse transcription-PCR (qRT-PCR)

Human *TNS3* gene expression in the fibroblasts was analysed by quantitative RT-PCR (qRT-PCR) using the ABI PRISM 7700 Sequence Detection System (Applied Biosystems, Warrington, UK). Different sets of fluorescent primers and probes for *TNS3* and a housekeeping gene (*GAPDH*) were used for multiplex real-time PCR detection of expression (Integrated DNA Technologies, Leuven, Belgium). Total RNA was first extracted from the fibroblasts using a kit (Qiagen, Crawley, UK), and subsequently reverse transcribed in a reaction mix containing oligodT primers, dNTPs, RT buffer, RNase inhibitor and reverse transcriptase (Fermentas, St. Leon-Rot, Germany). The resulting cDNA (5 μl) was then added to a qPCR reaction mix containing *TNS3* and *GAPDH* primers and probes, and qPCR polymerase master mix (Maxima, Fermentas) to a final volume of 20 μl. The cycling conditions were as described previously [8]. Each measurement was performed in triplicate wells per reaction. The relative standard curve method of quantitation was employed, using a standard curve of *TNS3* expression in cDNA from the human glioma cell line SNB-19.

### SDS-PAGE and western blot

Fibroblasts at near confluence in 6-well tissue culture plates were rinsed in ice-cold phosphate-buffered saline (PBS) and lysed in ice-cold lysis buffer composed of 1% NP-40, 1% deoxycholate, 5 mM EDTA, 1 mM EGTA in PBS, pH 7.4, supplemented with a protease inhibitor cocktail (Calbiochem, Nottingham, UK). Cell lysates were clarified by centrifugation at 20,800 *rcf* for 30 min at 4°C, from which the supernatants were transferred to new tubes. Total protein concentration was measured with using a bicinchoninic acid (BCA) assay kit (Sigma, Gillingham, UK). Equal protein amounts were loaded onto 10% polyacrylamide gels for reducing SDS-PAGE, after which proteins were transferred onto a PVDF membrane. Membranes were blocked in 5% milk powder in 25 mM Tris, 150 mM NaCl, 0.05% Tween-20, pH 8.0, and then probed with primary antibodies at optimal dilutions. The anti-Tensin3 antibodies used included two in-house rabbit polyclonal antibodies, raised against the N- and C-terminal regions of Tensin3, the C-terminal antibody having been characterised previously [8]. Also, two new commercial anti-Tensin3 polyclonal antibodies were used, raised in goat (Santa Cruz Biotechnology, CA) and rabbit (Sigma). Furthermore, a goat polyclonal ExoC6B antibody (Santa Cruz) was also tested on lysates. Membranes were incubated with the primary antibody overnight at 4°C, then washed 3 times for 5 min in wash buffer, 0.1 M Tris–HCl, 0.15 M NaCl, 0.05% Tween 20, pH 8.0. They were then incubated for 1 h at room temperature with secondary antibodies against the relevant species primary antibody, conjugated to horseradish peroxidase (HRP) (Promega, Southampton, UK). Further washes were followed by chemluminescent development using an H_2_O_2_ and luminol-based reagent mixture (HRP, Sigma).

### Immunoprecipitation

For immunoprecipitation (IP) experiments, fibroblasts were seeded into 10-cm tissue culture dishes and grown to near confluence before lysis. Cells lysates were prepared as for SDS-PAGE described above, and protein concentration was determined so as to prepare samples containing 500 µg total protein. Before IP, lysates were initially pre-cleared by incubation with with 10 μl protein A/G-agarose beads (Alpha Diagnostics, San Antonio, TX) and mixed with gentle end-to-end rotation for 1 h at 4°C. The beads were sedimented by quick centrifugation, and the supernatant collected into separate tubes to which 3 μg IP antibody was added; tubes were rotated overnight at 4°C. The next day, the beads were washed 3 times with ice-cold lysis buffer and once with PBS. The proteins bound to beads were removed by addition of 3x SDS loading buffer and brief boiling before being subjected to 10% SDS-PAGE and western blot for Tensin3 detection, as described above.

### Immunofluorescence staining and confocal microscopy

Fibroblasts were plated onto glass coverslips and allowed to adhere overnight at 37°C, 5% CO_2_. The next day, cells were rinsed with PBS and fixed with 4% formaldehyde for 15 min, and permeabilised with 0.2% Triton X-100 for 10 min. For staining, cells were first blocked for 1 h with 10% horse serum in PBS and then incubated with the primary antibody (rabbit anti-Tensin3 in 1.5% serum) for 2 h at room temperature. Cells were washed three times with PBS and then incubated with donkey anti-rabbit secondary antibodies conjugated to Alexa Fluor® 647, at 1:1000 dilution in 3% serum for 1 h at room temperature. The coverslips were washed three times with PBS and mounted onto glass slides using an aqueous, non-fluorescent mountant (Polysciences, Eppelheim, Germany). The samples were analysed under a confocal laser-scanning microscope (LSM710; Zeiss, Oberkochen, Germany), using a Plan Apochromatic 63× DIC oil objective (NA1.4). Images were processed with the software Zen2008 Light Edition (Zeiss). Specificity of the staining was verified by lack of fluorescence in cells incubated with secondary antibodies alone.

Furthermore, live cells in culture were also viewed under phase contrast microscopy, and images were captured using a CCD digital microscope camera (DP50, Olympus).

### Scratch-wound assay and live cell imaging microscopy

Fibroblasts were seeded in equal numbers (1.5 × 10^4^) into wells of a 24-well tissue culture plate and left to adhere overnight at 37°C and 5% CO_2_ prior the experiment. Cells were at 90% confluence the next day, at which point they were pre-incubated for 1 h with the live cell fluorescent dye Cell Tracker ™ Blue (Life Technologies, Paisley, UK). In each well, three straight, parallel scratches were made across the diameter of the well using a 0.58 mm orifice size pipette tip. From that point on, filling of the wound by migrating cells was monitored under a live cell (time-lapse) imaging system comprising a Zeiss Axiovert 200 M microscope (Carl Zeiss, Welwyn Garden City, UK) fitted with an incubator (37°C, 5% CO_2_, humid atmosphere). A 5x (air) objective was used; fluorescent images were acquired using Volocity software (v5.4; Perkin Elmer, Bucks, UK) such that 3 points across separate wound sections in each well were imaged every 1 h for 72 h to measure wound closure. The images were collated and movie sequences generated. Image analysis of cell movement was carried out using the free image processing program ImageJ (http://rsb.info.nih.gov/ij/). Experiments were performed in triplicate.

### Statistical analyses

The levels of Tensin3 mRNA, the rates of cell migration and proliferation at specific time points, and cell width in control vs proband fibroblasts were compared by unpaired *t* test. A *P* < 0.05 was considered statistically significant.

## Results

### Quantification of normal Tensin3 mRNA expression in proband and control fibroblasts

**Figure 1:**
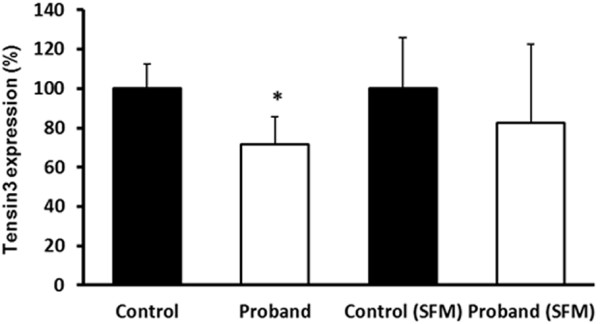
**Expression of Tensin3 mRNA expression in proband and control fibroblasts.** qRT-PCR was performed on RNA from cells maintained under both full growth medium (first pair of bars) as well as serum-free medium (SFM; second pair of bars). Results are presented as % ± SD expression normalised to housekeeping gene expression (ng *TNS3*/ng *GAPDH*). *P < 0.05 vs. control fibroblasts under full growth medium conditions; n = 3.

The levels of *TNS3* gene expression were quantitatively determined by real time qRT-PCR of RNA from proband and control fibroblasts under both serum-containing and serum-free conditions (Figure 1). In both conditions, the Tensin3 mRNA expression level in proband fibroblasts was 20-30% lower than in control cells, although the difference was statistically significant only in full growth medium conditions. This suggests that the chimeric mRNAs in the proband cells are less stable and at least partially degraded, as compared to the wildtype mRNAs for each gene in the control cells.

### Full length Tensin3 protein expression is lower in proband cells

**Figure 2:**
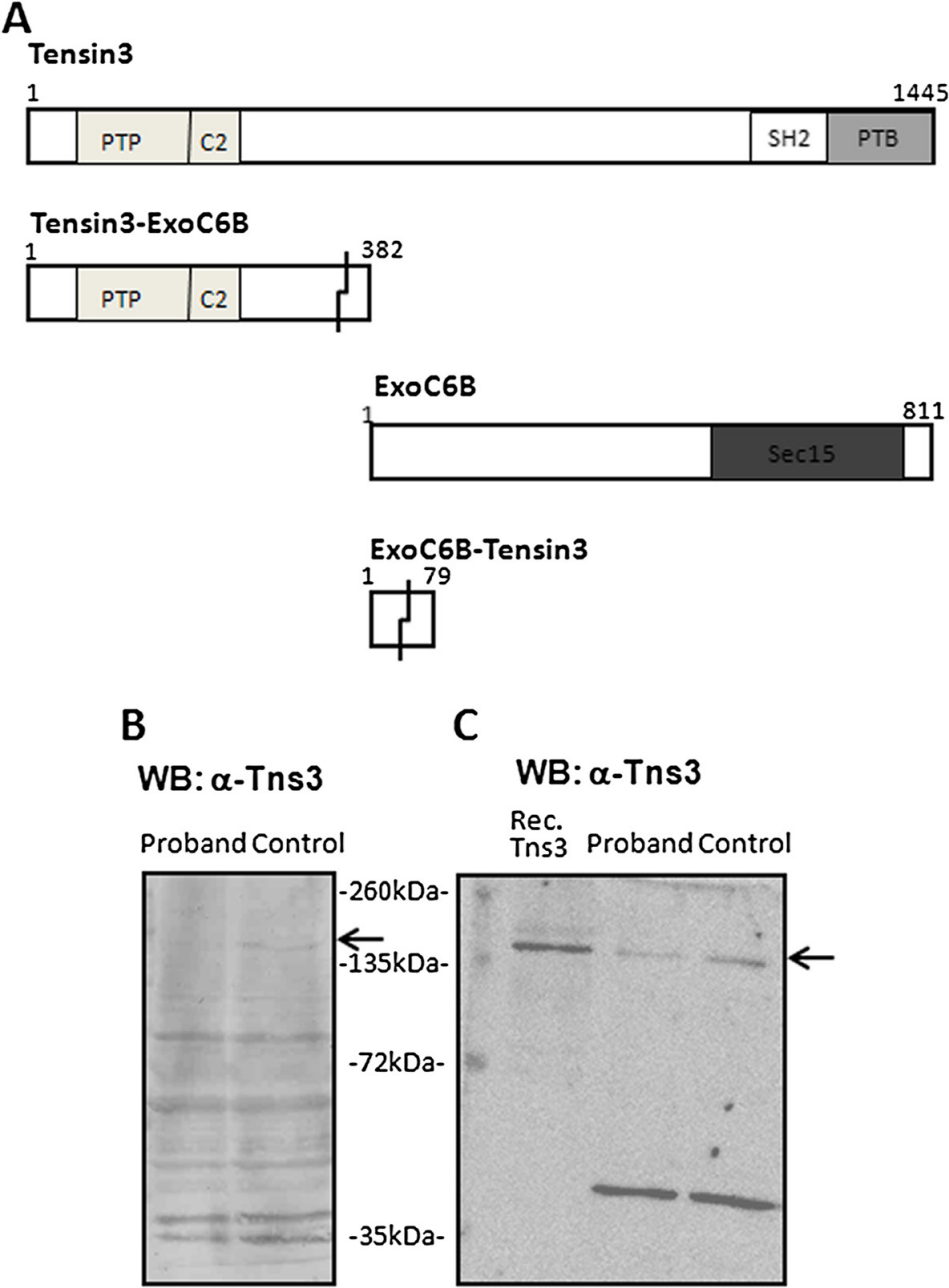
**Characterisation of Tensin3 protein expression in proband and control fibroblasts. A:** Schematic representation of wildtype Tensin3 and ExoC6B proteins, as well as putative chimeric proteins that could arise from unimpeded expression of the two new open reading frames created from the chromosomal translocation. Amino acid number is shown at the N and C terminal ends and identified protein domains are indicated in boxes within each protein. In the chimeric proteins, jagged lines indicate the fusion point of the wildtype sequence with that of a novel open reading frame. **B, C:** Western blots of Tensin3 expression in proband and control fibroblasts. Equal amounts of total protein from each cell extract were run on SDS-PAGE. Blots show immunoreactivity of two different anti-Tensin3 antibodies recognising the Tensin3 N-terminal, Santa Cruz (**B**) and Sigma (**C**). The Tensin3 band is indicated by an arrow. In blot c, lane 1 contains HEK 293 cell extract expressing recombinant human Tensin3 as a positive control. All blots are representatives of three separate western blots performed.

In the proband’s cells, the potential existed for expression of Tensin3-ExoC6B or ExoC6B-Tensin3 chimeric proteins, should their transcripts have been translated. Table 1 outlines the molecular compositions of all four possible protein products in the proband’s cells, also illustrated schematically in Figure 2A. The Tensin3-ExoC6B fusion protein would be composed of the first 340 amino acids of Tensin3 protein and the remaining 42 amino acids would be encoded by out-of-frame codons arising from the fusion with the *EXOC6B* sequence. This would give rise to a predicted molecular weight of 43 kDa on SDS-PAGE (*Sequence Analysis* program, Informagen, USA). The ExoC6B-Tensin3 fusion would be composed of the first 37 amino acids of ExoC6B and a remaining stretch of 42 amino acids with no identity, due a frameshift being introduced at the breakpoint junction. This would give rise to a predicted molecular weight of 9 kDa on SDS-PAGE.

**Table 1:** Molecular compositions of protein products of potential transcripts in the proband cells

**Protein (GenBank ID)**	**Amino acid composition**	**Approximate predicted molecular weight (kDa)**
Tensin3 (NP_073585)	1445 aa	155
ExoC6B (NP_056004)	811 aa	94
Tensin3-ExoC6B chimera	382 aa (aa 1–340 of Tensin3; aa 341–382 unique)	43
ExoC6B-Tensin3 chimera	79 aa (aa 1–37 of ExoC6B; aa 38–79 unique)	9

We carried out western blots to determine the expression level of endogenous Tensin3 protein in the proband’s fibroblasts versus those of an age-matched control. For this, we used several different antibodies against human Tensin3, including an in-house rabbit polyclonal that we have verified before [8], as well as commercial rabbit and goat polyclonal antibodies. To verify antibody specificity, equal amounts of protein for each sample were run on SDS-PAGE together with lysates from HEK293 cells stably expressing recombinant human Tensin3, as described previously [8]. The specificities of the antibodies varied, although in all western blots, a band was observed for Tensin3 at the expected molecular weight of around 170 kDa (Figure 2B, C), the same as for the Tensin3–transfected control sample (Figure 2C). In all cases, the Tensin3 immunoreactivity was significantly lower in the proband’s cell extracts as compared to those of the age-matched control cells. The most specific antibody was the commercial rabbit polyclonal, which showed a single clear band of the expected size for Tensin3, as well as a single non-specific band at around 50 kDa (Figure 2C). From this blot, densitometric analysis was used to measure the intensity of the Tensin3 band as normalised against the non-specific 50-kDa band for each sample. The normalised intensity values (100% for the 50-kDa band) were 25.44% (proband) and 42.31% (control), showing that the proband cells contain roughly half of the full length Tensin3 protein that control fibroblasts do.

For detecting the putative Tensin3-ExoC6B fusion protein, both the in-house and commercial rabbit anti-Tensin3 antibodies were raised against sequences in the N-terminal of Tensin3, and so would be expected to detect such a chimeric protein if it existed. However, no clear unique band in the region of 43 kDa was detected in the proband fibroblast extract using these antibodies (Figure 2B, C). In addition, we performed immunoprecipitation experiments on the proband cell extracts using an in-house N-terminal antibody to pull down both full-length Tensin3 as well any potential chimeric protein. However, neither full-length Tensin3 nor a specific 43-kDa band representing the Tensin3-ExoC6B fusion protein were detected in western blots of the immunoprecipitated complexes, indicating that the antibody failed to pull down either form of Tensin3 (not shown). Furthermore, a commercial antibody against ExoC6B was also tested on cell lysates, where a 94-kDa band for the full length protein was expected to be detected. However, no such band was visible for either sample (data not shown).

### Distinct morphology and immunofluorescence localisation of Tensin3 in proband fibroblasts

**Figure 3:**
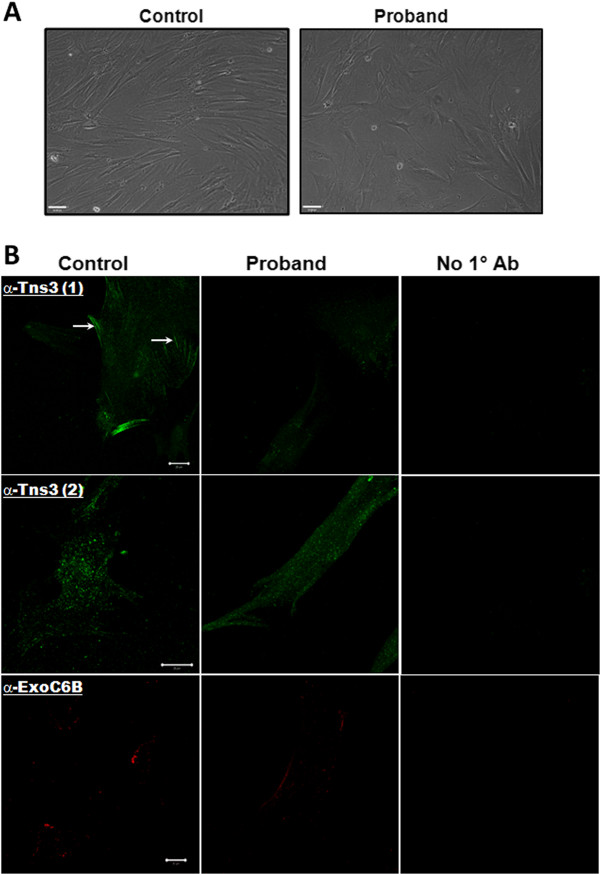
**Immunofluorescent confocal microscopic analysis of Tensin3 and ExoC6B in proband and control fibroblasts. A:** Live cells were imaged using phase contrast microscopy; scale bar represents 63 μm. **B:** Cells were fixed, permeabilised and stained using specific antibodies: anti-Tensin3 in-house antibody no. 1 (recognising a C-terminal sequence; green fluorescence; top row), anti-Tensin3 in-house antibody no. 2 (recognising an internal sequence; green fluorescence; middle row), and a commercial goat anti-ExoC6B antibody (red fluorescence; bottom row). The secondary antibody was coupled to Alexa Fluor® 647 fluorochrome. Stained cells were imaged under a confocal microscope as described. Arrows indicate Tensin3 immunoreactivity in actin filamentous structures. Negative control images were also taken of cells not incubated with primary antibody (third image each row). Scale bar represents 20 μm.

When viewed by phase contrast microscopy, proband fibroblasts exhibited a distinct morphology as compared to control cells. Proband cells appeared shorter and broader in cell body dimensions, whilst control fibroblasts appeared longer and thinner (Figure 3A). We quantified the width of the cell bodies within each culture (ImageJ) and determined the proband cells to be significantly wider than control fibroblasts (mean ± SEM width (arbitrary units) of proband cells 107.23 ± 3.62, control cells 65.36 ± 1.94; *P* < 0.001 (n = 28)). Also, immunofluorescent staining and confocal microscopic analysis were carried out in proband and control fibroblasts to detect Tensin3 and ExoC6B expression. Using two separate in-house antibodies, staining for Tensin3 was observed in both types of cells at similar intensities, mostly exhibiting a punctate pattern of expression throughout the cytoplasm (Figure 3B), as has previously been reported by us for Tensin2 [14]. However, in control fibroblasts alone, Tensin3 staining was also apparent along cytoskeletal filaments and the cell periphery, whereas this profile was absent in proband cells. Cells were also immunostained for ExoC6B, with both cell types exhibiting a weak punctuate and membrane-associated fluorescence pattern in equal measure. All immunofluorescent staining was verified by omission of primary antibody in negative control samples, where negligible fluorescence was observed.

### Proband fibroblasts exhibit a greater migration rate as compared to control cells

**Figure 4:**
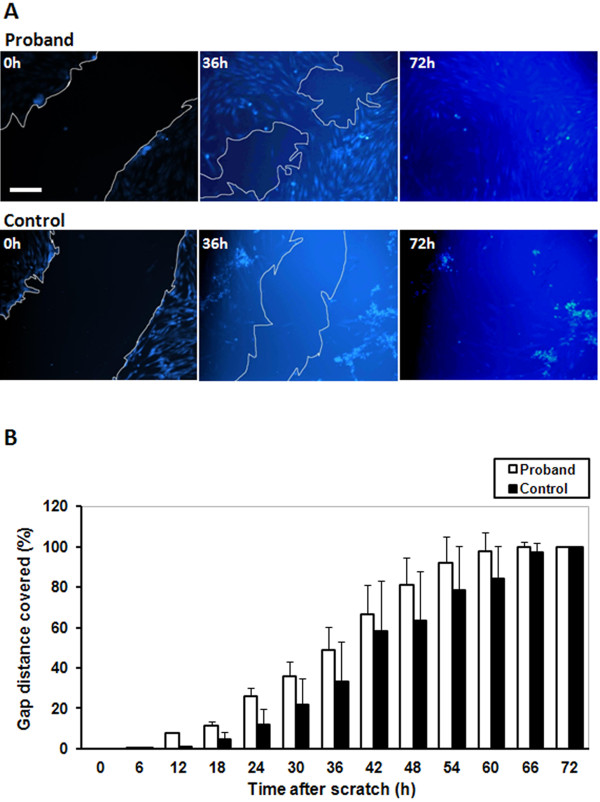
**Cell migration assay of proband and control fibroblasts.** Live cell imaging was used to monitor movement of cells into an open scratch wound over 72 h, with images being captured every 1 h. **A:** Image snapshots were captured at 0, 36 and 72 h after post-wound image capture began. Live cells have been stained with Cell Tracker^™^ Blue fluorescent dye, and a white line marks the edge of the scratch wound. Scale bar represents 200 μm. **B:** Quantitative representation of distance covered by cells within the scratch wound over the 72-h analysis period. Results are expressed as mean ± SEM % distance covered by the cells per unit time (h), with 100% representing complete filling of the wound; n = 3 scratches for each cell type.

The migration rates of proband and control fibroblasts were measured using a scratch wound assay, with cells being monitored in real time under live cell imaging (Figure 4). Additional files 1 and 2 contain video sequences showing scratch wound filling by the two cell cultures over 72 h, whilst Figure 4A shows snapshots at 0, 36 and 72 h after post-wound image capture began. Quantification of the results showed that proband fibroblasts migrated at a higher rate than control cells over the entire 72 h (Figure 4B). Proband fibroblasts first began to migrate out from the periphery of the wound at 12 h after its creation, versus 18 h for control cells. Proband cells showed consistently greater coverage of the wound area than control cells between 12 h and 60 h, with complete closure of the wound being achieved by 60 h for proband cells versus 72 h for control cells. When considering the rates of migration (distance covered per unit time), proband cells showed statistically significantly higher migration rates than control cells within the initial 24-h period (*P* < 0.001; n = 3), afterwards being caught up by control cells due to nearing confluence in the wells. Furthermore, cell proliferation played no part in the observed cell migration differences, as the two cell cultures exhibited similar proliferation rates with no significant differences (Additional file 3).

## Discussion

In the present study, we have built upon the initial discovery of a unique cytogenetic abnormality in a young patient that constituted a reciprocal chromosomal translocation, t(2;7) (p13;p12), involving the genes *TNS3* and *EXOC6B*[12]. However, at that time no adequate tools were available to study the status of the proteins that may be affected. Therefore, in the present study, we have utilised a set of new and unique tools specific for Tensin3, to carry out a molecular and functional characterisation of cells harvested from the patient. The major finding from our study is that wildtype Tensin3 expression is impaired in the patient, the functional significance of which is translated to a greater migratory capacity in patient’s cells. These data tie in well with our previous observations and ensuing hypothesis that Tensin3 normally functions as an anti-migratory molecule, which is supported by our previous observation of a loss of Tensin3 in human renal cell carcinoma amongst other cancers [8]. Furthermore, the important functional role of Tensin3 in the kidney is underscored by the fact that the patient under study showed impaired kidney development and function.

Our qRT-PCR analysis revealed a lower expression of normal Tensin3 mRNA transcripts in proband cells as compared to control cells. This was significant however only in full growth medium, possibly due to the fact that Tensin3 expression was much lower under starvation conditions to barely detectable levels to be able to discern a clear difference (not shown). Provided that correct splicing of pre-mRNA occurred in all transcript forms, the qRT-PCR primers and probes we used would recognise both wildtype and chimeric *EXOC6B*-*TNS3* transcripts. These quantitative expression results therefore show that there is an imbalance in the levels of transcripts containing the Tensin3 sequences analysed. This indicates that the cellular quality control mechanisms have removed at least a proportion of the aberrant transcripts e.g. through nonsense-mediated mRNA decay. The chimeric and *EXOC6B* transcripts were not measured in this study due to lack of specific primers/probes.

At the protein level, we found a lower basal expression of endogenous wildtype Tensin3 in the proband cells. However, we observed no concomitant presence of either of the two putative chimeric fusion proteins. In particular, we utilised a set of Tensin3 N-terminal antibodies that would have detected the Tensin3-ExoC6B chimera as a 43-kDa band on western blots. A possible explanation for the absence of the chimeric protein could be that the fusion DNA sequence was not transcribed. Alternatively, both transcription and translation may occur but may have been followed rapidly by post-translational processing and degradation of the protein, driven by the cell’s ubiquitin-proteasome complex [15]. However, the most likely event is, as premature translation-termination codons (PTCs) are produced in the case of both fusion sequences, the selective recognition and degradation of the aberrant transcript prior to translation, occurring through the nonsense-mediated mRNA decay (NMD) pathway [16]. The occurrence of PTCs upstream of a fixed point in the penultimate exon of the normal gene sequence enables them to be distinguished from *bona fide* termination codons. The NMD pathway then degrades these transcripts through endo- or exonucleolytic cleavage, as part of a ubiquitous mechanism to prevent cellular production of potentially toxic truncated proteins, which is moreover linked with the proteasome system [15].

Microscopic analyses of the proband and control cells revealed subtle differences between them in terms of their morphologies and the distribution of intracellular Tensin3 expression. Proband fibroblasts cells exhibited significantly broader and shorter cell bodies than control cells, which could arise from a lesser tension due to reduced contacts between the cytoskeleton and the cell periphery. To probe this, it will be worthwhile as a follow-up to analyse molecular markers of cell shape changes, including focal adhesion and mesenchymal proteins. Furthermore, immunofluorescent staining for Tensin3 showed a punctate expression pattern within both cell types with equal intensities and distributions. These could display fibrillar adhesions, which is where Tensin3 was recently observed to mainly localise to in human skin fibroblasts, colocalising with α5β1 integrin within those structures [17]. However, an additional clear and well-defined Tensin3 staining along cytoskeletal filaments and the cell periphery was present only in the control cells. Therefore, a lower amount of full-length Tensin3 protein appears to exist in the proband cells, as a sufficient expression level allows a subset of wildtype Tensin3 to closely interact with actin filaments. This feature would be plausible as the larger Tensin family members possess homologous actin-binding domains in their N terminal regions, which the shorter Tensin4 (cten) lacks [6].

Another phenotypic difference we observed between proband and control fibroblasts was the greater migration rate of proband cells. We have previously shown that Tensin3 overexpression in HEK 293 cells inhibits cell migration, and this correlates with downregulation of Tensins in human kidney cancer [8]. Furthermore, EGF treatment of cancer cells leads to downregulation of Tensin3 and concomitant upregulation of cten, correlating with enhanced cell migration [11]. These results therefore indicate that Tensin3 normally functions as a cell migration-inhibitory molecule. At least in cancer cells, Tensin3 appears to control cell migration by signalling through the deleted in liver cancer-1 (DLC-1) protein, which is a Rho-specific GTPase activating protein (Rho-GAP) [5]. In this pathway, Tensin3 activates the Rho-GAP activity of DLC-1, enabling it to inhibit RhoA through hydrolysis of its bound GTP, and consequently Rho-kinase (ROCK), the immediate down-stream effector of RhoA [18]. This results in reduced cell motility due to a reduction in stress fibre formation and dissociation of focal adhesions. However, it must also be stated that Tensin3 has also been shown to promote cancer cell migration and growth, in particular through Src-dependent tyrosine phosphorylation of its SH2 domain and subsequent specific interactions [5]. This feature however appears to be an outcome in cancer cells, where DLC1 is downregulated and Src oncogene activity is high. Therefore, in the skin fibroblasts that we have studied here, where the opposite is true, normal expression of wildtype Tensin3 appears have the expected effect.

As gain-of–function expression of putative chimeric proteins is unlikely, we therefore propose that Tensin3 haploinsufficiency is a pathogenetic mechanism for the cellular phenotypes that we have observed. These phenotypic changes may play a role in the syndromic status of the patient reported in the original study, which included impaired kidney development and function, recurrent pulmonary infections, severe gastroesophageal reflux, bone marrow hypoplasia, and retarded development [12]. Although speculation is limited, Tensin3 impairment in humans may at least contribute to a kidney phenotype, as Tensin3 is normally expressed in the human kidney cortex, being mainly localised to proximal tubular epithelial cells [8]. Moreover, genetic impairment of Tensin3 may have a wider consequence than simply affecting Tensin3 and ExoC6B proteins only, as we previously showed that the cellular distribution of Tensin1 is also reduced in the Tensin3-haploinsufficient fibroblasts of the patient [12]. Therefore, it may be that the concurrent loss of more than one Tensin family member is the true driving force behind the phenotypic alterations observed in the patient. Interestingly, a separate study has identified a different balanced translocation involving the *TNS3* gene, in this case also affecting the *FGFR1* gene, with the patient displaying hypogonadotropic hypogonadism and cleft lip and palate [19]. However, no unique phenotypic abnormalities were observed in that patient that could distinguish them from those seen in Kallmann Syndrome arising from *FGFR1* point mutations. Therefore, although heterozygous Tensin3 impairment did not seem to have a noticeable effect in that case, the possibility could not be excluded that the predicted fusion proteins were compensating through functioning as the normal protein. It is noteworthy that more than one type of translocation involving chromosome position 7p12.3 has been detected, indicating that this location is more susceptible to breakage than most other locations [20]. Furthermore, Tensin3 knockout mice display growth retardation and postnatal lethality, as well as incomplete development of the lung, small intestine and bone, although heterozygous mice were phenotypically normal [21]. Therefore, Tensin3 appears to play a role in the development and function of several organs of the body, through maintenance of cell and tissue architecture. In contrast to Tensin3, the precise function and role of the ExoC6B protein are barely characterised, partly due to a lack of reliable and specific tools to study this molecule. Therefore, the functional consequences of the chromosomal translocation effect due to changes in ExoC6B protein remain to be investigated.

## Conclusions

Tensin3 haploinsufficiency manifests itself in an altered phenotype in the explanted cells of the patient, involving reduced levels of wildtype Tensin3 protein as well as an enhanced migration rate. This novel information highlights the consequences of Tensin3 impairment in human pathophysiology, as well as elucidating further the role of Tensin3 in normal human physiology.

## Competing interest

The authors confirm that there are no competing interests.

## Authors’ contributions

SH, SB and GB conceived of and designed the study. SB and GB created the cell cultures. DL and SH performed the cell culture, microscopy and protein expression analysis. DL and JC performed the gene expression analysis. DL and JRS performed the cell migration experiments. All authors read and approved the final manuscript.

## Pre-publication history

The pre-publication history for this paper can be accessed here:

http://www.biomedcentral.com/1471-2350/14/65/prepub

## Supplementary Material

Additional file 1 **The progress of representative cell migration experiments on control (Additional file 1; Control.mpg) cells.** Confluent control and proband skin fibroblasts were rendered fluorescent with a tracker dye, and subjected to a scratch wound at time 0. Cells were kept under normal culture conditions during the experiment. From that point on, single microscope images were acquired every 1 h for 72 h, from which the movie sequences are derived. Click here for file

Additional file 2 **The progress of representative cell migration experiments on proband (Additional file 2; Proband.mpg) cells.** Confluent control and proband skin fibroblasts were rendered fluorescent with a tracker dye, and subjected to a scratch wound at time 0. Cells were kept under normal culture conditions during the experiment. From that point on, single microscope images were acquired every 1 h for 72 h, from which the movie sequences are derived. Click here for file

Additional file 3 **Cell number was determined on days 3 and 5 by cell trypsination and counting in a haemocytometer.** Bars represent mean cell number (% relative to Day 0) ± SEM (n = 3 separate experiments in quadruplicate wells); NS, no significant difference between proband and control cell numbers on days 3 and 5; *t* test. Click here for file
