# Supplementary material for: Functional characterisation of human cells harbouring a novel t(2p;7p) translocation involving TNS3 and EXOC6B genes
Source: BMC Med Genet. 2013 Jun 28;14:65. doi: 10.1186/1471-2350-14-65 (PMC3728010; doi:10.1186/1471-2350-14-65)
Supplement: Additional file 3 — Cell number was determined on days 3 and 5 by cell trypsination and counting in a haemocytometer. Bars represent mean cell number (% relative to Day 0) ± SEM (n = 3 separate experiments in quadruplicate wells); NS, no significant difference between proband and control cell numbers on days 3 and 5; t test. [file 1471-2350-14-65-S3.docx]

**Additional file 3** (Ludwig *et al*. 2013).

**Additional file 3.** Cell proliferation assay. Equal numbers of proband and control fibroblasts were seeded in a 96-well plate (day 0), then incubated for up to 5 days. Cell number was determined on days 3 and 5 by cell trypsination and counting in a haemocytometer. Bars represent mean cell number (% relative to Day 0) ±SEM (n=3 separate experiments in quadruplicate wells); NS, no significant difference between proband and control cell numbers on days 3 and 5; *t* test.
